# Concept, Influencing Factors, and Interventions of Nursing Health Leadership: A Scoping Review

**DOI:** 10.1155/jonm/5212844

**Published:** 2025-02-15

**Authors:** Jing-jing Zhao, Ying Shen, Lian-hong Li, Jing-ying Zhang, Min-xing Ou, Xiu-jie Zhang, Tie-ying Shi

**Affiliations:** ^1^Nursing Department, First Affiliated Hospital of Dalian Medical University, Dalian, Liaoning, China; ^2^School of Nursing, Dalian Medical University, Dalian, Liaoning, China

**Keywords:** health awareness, health behavior, health leadership, health value, nursing leader, scoping review, well-being

## Abstract

**Background:** With increasing burnout and turnover rates among nurses, health leadership can effectively improve the health and well-being of both leaders and staff. However, in the nursing field, the definition and mechanisms of health leadership remain unclear and require further research.

**Aim:** The main objective of this study was to define the concept of health leadership in nursing, construct a nursing model program, and promote the formation of normative nursing health leadership theories and strategies.

**Evaluation:** We searched the PubMed, CINAHL, Embase, Web of Science, PsycINFO, and Scopus databases. The following themes were extracted from the included articles: the concept, assessment tools for health leadership, influencing factors, intervention measures, and health effects.

**Key Issues:** A systematic search of the relevant databases yielded 3161 initial search results. Thirty-two studies were eligible for inclusion. Research shows that the concepts and measurement tools of health leadership are rarely applied in the field of nursing. Health leadership is influenced by personal factors (e.g., leadership qualities such as care, support, courage, patience, tolerance for uncertainty, persuasion skills, work ethic, pressure, experience, effort–reward imbalance, etc.; specific knowledge; self-awareness; psychological capital; leadership style; motivation; consideration; and a commitment to health issues) and organizational factors (e.g., work environment, attention to subordinates, job expectations, and relationship transparency). Several studies have demonstrated that interventions, such as personal development planning, leadership training, face-to-face communication, self-directed learning, and reflection, are effective in promoting healthy leadership styles and improving the well-being of leaders and employees.

**Conclusions:** We constructed a health leadership model as a reference for the development of relevant measurement tools and intervention strategies for the nursing field.

**Implications for Nursing Management:** Nursing leaders should focus on their health and that of their subordinates, develop and implement health leadership, and aim for improvement in employees' well-being and nursing quality.

## 1. Introduction

Currently, with the increasing global demand for health workers, a net shortage of 15 million global health workers is anticipated by 2030 [1]. An assessment of the supply and demand for registered nurses in the United States from 2016 to 2030 showed that the nursing workforce is facing a persistent shortage [2]. Staffing deficiency, low job satisfaction, less nursing experience, and younger ages are important reasons for nurses' absenteeism and turnover intentions [3]. Stress and leadership issues also seriously impact nurses' dissatisfaction and turnover intentions [4]. If nursing leaders show toxic leadership behaviors such as ignoring, unfair treatment, self-centeredness, excessive pressure, and work in action, nurses will have lower job satisfaction and be more willing to leave the nursing profession [5]. With the development and application of leadership, the theory of leadership has been widely applied in different fields. Many studies have shown that leadership has a positive influence on employees' health, satisfaction, and job performance [6–8]. For example, health promotion leadership focuses more on the health of leaders and employees and is more meaningful in terms of employees' health, happiness, work attitude, and innovative work behaviors [9]. The core element of health promotion leadership is a leader's health management style or a series of behaviors that promote the health of organizational members [9]. Leaders are committed to promoting activities related to organizational health, manifesting supportive leadership styles, and developing health-promoting workplaces [9]. To provide employees with sufficient work resources for balancing work demands, caring about their health, and encouraging them to develop a healthy lifestyle are crucial [10]. However, some leadership styles are task-centered (e.g., discordant, instrumental, and managerial), which concentrate on completing tasks to achieve high nursing quality but generally reduce nurses' job satisfaction [11]. Passive avoidance and free-ranging leadership styles also increase nurses' working stress and expected turnover rate due to their attitude of avoiding responsibility and participation [4, 12]. Compared with these types of leadership styles, health leadership not only focuses on skill development but also emphasizes the consequence of health, which is conducive to the growth and health well-being of nurses, and further improves patient satisfaction [13]. In addition, previous studies did not limit health leadership to formal leaders but also applied to informal leaders [9, 14]. Therefore, the scope of health leadership is relatively wide, and its application in the field of nursing has practical significance for the health and well-being of nursing leaders, nurse staff, and patients [13].

There are numerous international studies on the application of health leadership in different professions, all of which emphasize the role of healthy and beneficial leadership on the positive health of subordinates [9, 14, 15]. However, the definition of health leadership in the field of nursing is not clear, so it is necessary to further clarify the concept of health leadership in the field of nursing [9]. In addition, there are many studies evaluating factors related to health leadership; for example, the critical preconditions of healthy leadership are personal characteristics and organizational health culture [15], whose existence enables leaders to conduct behaviors that promote employee health [9]. Frangieh and Jones also emphasized this point in their study on nursing managers [16]. These relevant factors can provide a reference for a more comprehensive understanding of health leadership [17]; however, there is no consensus in the literature to consider the factors that influence health leadership in the nursing profession [16]. Many studies have developed assessment tools for health leadership, such as the Health and Development Promotion Leadership Behavior Questionnaire (HDLBQ) and the Organizational Health and Safety Scale, but they apply to a wide range of professions [9] and few assessment tools focus on health leadership in the field of nursing.

At present, there is a lack of unified opinions and experience to prove the influencing factors and assessment tools of health leadership in the nursing profession [18]. This study summarizes the scope of the application of health leadership in the nursing field, laying a foundation for further guiding the application and development of health leadership in nursing leaders.

## 2. Aim

With this scoping review, we aimed to define the concept of health leadership in the nursing field and review leadership assessment tools, influencing factors, and interventions to develop normative theories and strategies to promote health leadership among nursing leaders.

## 3. Methodology

### 3.1. Design

A scoping review is a form of knowledge synthesis; it can outline the core concepts of a field, identify research progress, discover the diversity of existing knowledge, and determine the direction of follow-up research by systematically searching, selecting, and synthesizing existing knowledge [19]. This study was conducted in strict accordance with the Preferred Reporting Item for Systematic Review and Meta-Analysis Extension for Scope Evaluation (PRISMA-ScR) reporting guidelines by Tricco et al. [20].

### 3.2. Search Methods

This study strictly follows the latest methodological guidelines for JBI scoping review proposed by Peters et al. in 2020 [21], which is aligned with PRISMA-ScR [20] to ensure consistency in reporting. The various concepts, measurement tools, and well-being of nursing employees are mapped through different stages (identifying research questions, identifying relevant studies, research selections, mapping data, collating, summarizing, and reporting results), and the relevant factors and interventions supporting the field of health leadership research are elucidated.

Based on the review of the existing literature, we have proposed four questions:

• What is the concept of health leadership in nursing?
• What tools do you use to measure health leadership?
• What are the influencing factors of nursing health leadership?
• What interventions can improve nursing health leadership?

### 3.3. Identifying Relevant Studies

**Figure 1:**
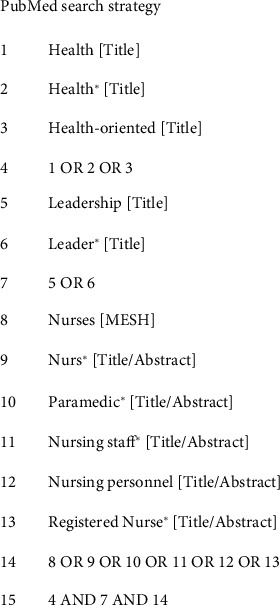
PubMed search strategy.

We reviewed relevant studies retrieved from the following computerized databases: PubMed, CINAHL, Embase, Web of Science, PsycINFO, and Scopus. A combination of subject headings and free words was used. The search strategy was determined by the project team based on the research questions. Search terms included the following: health, OR health⁣^∗^, OR health-oriented, AND leadership, OR leader⁣^∗^, AND nurses, OR nurses⁣^∗^, OR paramedics, OR nursing staff⁣^∗^, OR nursing personnel, and OR registered nurses⁣^∗^. We used the Boolean operators “AND” and “OR” to combine search terms. We reviewed the list of references for all included studies to identify possible additional papers. The search period ranged from the establishment of a database to 17 July 2023. The PubMed search strategy is shown in Figure 1.

### 3.4. Selecting the Literature

#### 3.4.1. Inclusion and Exclusion Criteria

The inclusion and exclusion criteria were determined following the PCC [21] principle, where P stands for the population (nursing leaders and nurses), the first C stands for the concept (in this study, the concept of health leadership), and the second C stands for context (the field of nursing).

Inclusive criteria are as follows:

• Nursing leaders and nurses.
• Mainly related to the concept of health leadership, influencing factors, intervention measures, evaluation methods, and tools.
• Published in English.
• All types of studies.

Exclusion criteria are as follows:

• Repeated inclusion.
• Unable to access the full text.
• Summary of the meeting.

### 3.5. Charting the Data

We imported retrieved literature into EndNote20 to delete duplicate studies. Based on the inclusion and exclusion criteria, two independent screening rounds were conducted by two researchers (J.-j.Z. and Y.S.). In the first round, the titles and abstracts of the articles were screened, and in the second round, the full texts of the remaining articles were screened to determine whether they met the inclusion requirements. For differences between researchers in the process of literature selection, a third researcher (X.-j.Z.) negotiated a resolution. Based on the purpose of the study, we extracted the contents of the included studies and sorted them into data charts. Data charts included the following: author, country of origin, type of study, sample size, research topic, assessment tools, relevant factors, interventions, and health effects.

## 4. Results

### 4.1. Bibliographic Overview

**Figure 2:**
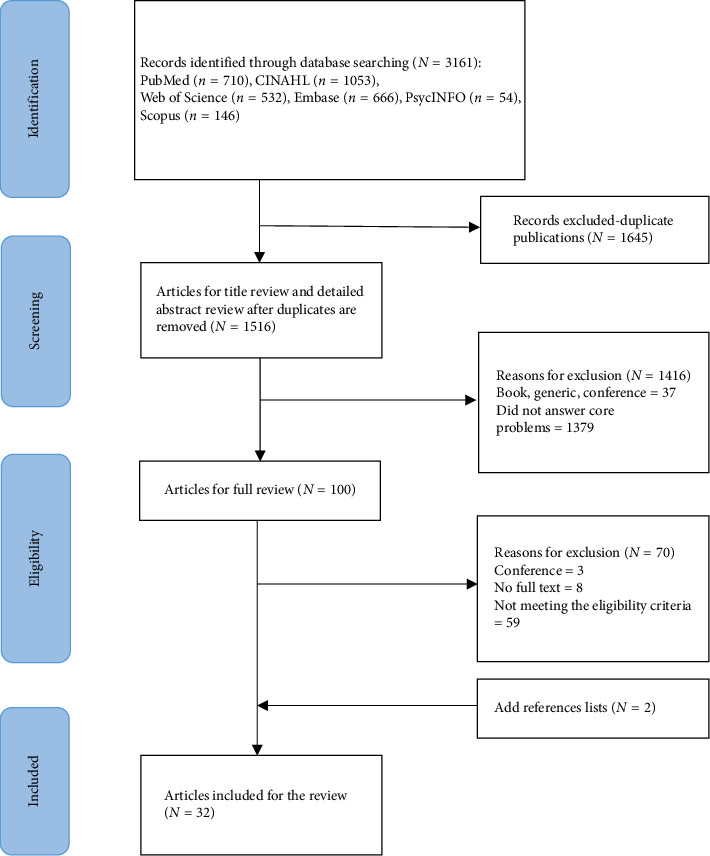
PRISMA flow diagram of the article selection process.

We retrieved 3161 articles from the electronic database search. After removing duplicates, 1516 titles and abstracts were screened, 100 full texts were evaluated, 70 full texts were excluded for various reasons, and two were included through the selection of references [22, 23]. This review included a total of 32 articles (see Figure 2). The basic information included in the literature is presented in Table 1.

### 4.2. Concepts Related to Health Leadership

This study found only seven articles related to health leadership in the nursing field (Table 2) [13, 18, 32, 34, 40, 44, 50], of which three were health-oriented leadership [32, 34, 40], three were health-promoting leadership [13, 18, 50], and one was healthful leadership [44]. Health-oriented leadership [32, 34, 40] includes both “leader-centered” and “staff-centered” aspects. Leader-centered aspects include leaders' health values, health awareness, and behaviors that influence leaders' own health behaviors and stress experiences, while staff-centered aspects include creating working conditions that promote mental health in participatory processes and direct, focused communication and interaction with workers [32, 34, 40]. Health promotion leadership [13, 18, 50] includes environmental characteristics (e.g., focus on engagement, meaningful work, and skill development) and personal leadership characteristics (e.g., being caring, supportive, courageous, responsible, attentive, and ethical), where the leader is committed to the health promotion of staff and is accountable for action, maintains open communication, and accommodates nurses to participate in the change process. Health promotion leaders maintain a special focus on health by taking responsibility for their health and the health of their employees [18]. Healthful leadership consists of six theories (being visible and present, being open and engaging, caring for self and others, embodying values, being prepared and preparing others, and using available information and support) that embody values such as compassion, honesty, openness, humility, respect, vulnerability, and courage [44]. Although the concept of health leadership is less applied in the field of nursing, the characteristics of health leadership have been found in other styles through studying other articles, thus making up for the lack of specific concepts of health leadership in the field of nursing (Table 2) [22, 24, 26, 28–31, 33, 38, 43, 45, 46, 49].

### 4.3. Health Leadership Assessment Tool

A total of 24 leadership assessment tools were included in this study (Table 3) [23–25, 27–31, 33, 35, 37–39, 43, 45–47, 49–51]. The Authentic Leadership Questionnaire (ALQ) [29–31, 33, 45, 49] and Multifactor Leadership Questionnaire (MLQ) [24, 25, 35, 39] were the most mentioned tools. The MLQ was originally developed as a tool to measure the transformational, active, and passive leadership attributes of leaders and subordinates [24, 25, 35, 39]. With constant revision, MLQ-5X Short Form has undergone rigorous psychological testing and is now considered the most effective measure of transactional and transformational leadership [28, 37]. The highest correlation was with Health-Promoting Leadership Conditions [50] (mentioned in only one article). The LMX 7 [28], ICU Nurse–Physician Questionnaire [35], Modified Nurse Manager Inventory Tool [35], Servant Leadership Behavior Scale [24], and SF-12v2 Health Survey [37] were not mentioned for reliability; however, the other scales all had reliability.

### 4.4. Factors Related to Health Leadership

In the literature review, six articles mentioning the influential factors of health leadership in the field of nursing were found [13, 18, 32, 34, 44, 50], and 13 articles mentioned the influential factors of other styles of leadership [22, 23, 25, 26, 30, 36, 37, 42, 45, 47–49, 51] (Table 2). These influencing factors have common ground and can be divided into personal factors and organizational factors. Personal factors include leadership qualities [18, 23, 32, 36, 42, 44, 47, 48, 50, 51] (e.g., care, support, courage, patience, tolerance for uncertainty, persuasion skills, work ethic, pressure, experience, and effort-reward imbalance), specific knowledge [32], self-awareness [42, 45], psychological capital [22], leadership style [37, 42, 49], consideration [18, 23, 25], a commitment to health issues [32], and motivation [25, 42] (e.g., intellectual motivation, motivation for a leadership role, and motivation of subordinates). In terms of organizational factors, key factors include the work environment [18, 26, 32, 48] (e.g., organizational culture, organizational vision, and focus on participation), attention to subordinates [13, 34], work expectations [25, 30] (e.g., workload, control, and reward), and relationship transparency [42, 45]. The health and well-being of nursing workers include both individual and organizational levels (Table 2). Individual levels include a focus on reduced burnout [22, 30, 36, 43, 45, 49, 50], increased satisfaction [22, 23, 29, 35, 36, 42, 43], well-being [28, 31, 37], mental health [22, 25, 29, 30, 34, 40, 47], reduced depersonalization [25], accomplishment [25], improved professional self-efficacy [30], emotional commitment [43], and a desire for creativity [33]. At the organizational level, the focus is promoting a healthy work environment [13, 24, 26, 29–31, 38, 46], encouraging employee engagement [35], and implementing effective processes [35].

### 4.5. Interventions for Health Leadership

In this study, there were two articles describing health leadership interventions [40, 44] (e.g., self-directed learning and reflection [40], online or face-to-face communication with nurses [44], mental health education [40], creating a learning space [44], and participation in nursing practices [40, 44]), two articles describe interventions for other styles of leadership [30, 35] (e.g., personal development plans [35], self-directed learning and reflection [35], online or face-to-face communication with nurses [35], and leadership training [30]), as shown in Table 2.

## 5. Discussion

This review aims to define the concept of health leadership in the nursing field and to examine leadership assessment tools, influencing factors, and interventions, to develop normative theories and strategies to promote health leadership among nursing leaders. To achieve this aim, the contents of 32 papers were analyzed and discussed. The results are as follows: (1) few papers reported the concept of health leadership, and some papers reported the concept of other styles of leadership, but they do include features of health leadership; (2) only one paper reported on the Health-Promoting Leadership Conditions, and some reported on other leadership style assessment tools; (3) some papers reported the health benefits of health leadership for nursing staffs; (4) some papers reported the background factors of health leadership (the basis for the further development of health leadership measurement tools in the nursing field); and (5) other papers reported interventions for health leadership that provide a basis for nursing managers to develop a health leadership model.

### 5.1. The Concept of Health Leadership

**Figure 3:**
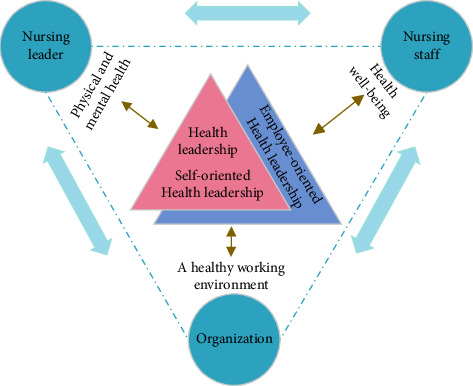
A conceptual model of health leadership in nursing.

Concepts of leadership are evolving, but they seem to be broad and not specific in terms of different population characteristics and situations [41]; the same is true for concepts of health leadership. Health promotion leadership, health-oriented leadership, and healthful leadership have similar conceptual characteristics, that is, nursing leaders actively participate in the health promotion of nursing employees, maintain communication and interaction, create a healthy working environment, and pay attention to their own and employees' physical and mental health through health values, health awareness and health behaviors [10, 13, 18, 32, 34, 40, 44, 50, 52]. Research shows that the attributes of healthy leadership come in the form of authenticity, reflecting values such as support, compassion, empathy, authenticity, openness, engagement, caring, and courage that create the conditions for leaders to build positive and healthy relationships between themselves, others, and their organizations [13, 18, 32, 34, 40, 44, 50]. Furthermore, other types of leadership (such as transformational leadership [28], authentic leadership [22, 24, 26, 29–31, 33, 38, 45, 46, 49], and ethical leadership [43]) are specific leadership styles, but their definitions and characteristics include health-related leadership attributes that have the effect of promoting employee health (for example, transformational leadership have high credibility, exemplary character, attractive vision, the ability to motivate employees, provision of support and development. Authentic leadership is transparent, optimistic, resilient, ethical, confident, and future-oriented. Ethical leadership has personal qualities, interpersonal relationships, selflessness, integrity, fairness, care and support for subordinates, and other leadership characteristics, as shown in Table 2). Transformational leadership reduces burnout and stress by motivating staff through an attractive vision, providing support and development for staff, and stimulating creativity and independent thinking in nurses [28]. Authentic leadership can influence employees' attitudes and creativity and add positivity through characteristics such as confidence, optimism, hope, and positive emotions [45]. When leaders are open and transparent to nurses and have a high degree of integrity, it will promote nurses' inclusiveness and form a healthy working environment, improve their job satisfaction, and have a positive impact on staff performance and organizational results [22, 24, 26, 29–31, 38, 46]. In addition, it will also reduce bullying at work and prevent nurses from burnout or developing poor mental health [22, 30, 45, 49]. Ethical leadership creates a positive and psychologically safe working environment through personal qualities, behaviors, and interpersonal relationships, such as fairness, caring, and supporting employees, and establishes a positive relationship with subordinates to improve employees' job satisfaction [43]. When employees are treated fairly, treated with respect, and supported when needed, emotional attachment, identification, and engagement with the organization also increase [43]. Although leaders shape organizational culture, these positive organizational cultures can also shape leaders and demonstrate more authentic leadership in an environment where leaders feel proud and satisfied [26]. Therefore, these types of leadership (such as transformational leadership, authentic leadership, and ethical leadership) also promote the health of leaders, employees, and organizations through their characteristics, thus providing a reference for health leadership in the nursing field [22, 24, 26, 28–31, 33, 38, 43, 45, 46, 49]. We developed a conceptual model of health leadership in nursing based on the concepts of health-promoting leadership, health-oriented leadership, and healthful leadership (Figure 3), in which nursing leaders, nursing staff, and organizations can interact and influence each other to continuously improve the overall health status of the individual and the organization (as shown by the arrows in the figure) [6, 26, 53]. By paying attention to the health of themselves and their employees, leaders with health leadership have a positive impact on nursing managers, nursing employees, and organizations, such as promoting the physical and mental health of leaders [10, 34, 40, 54], promoting the formation of a healthy working environment in the organization [13, 24, 26, 29–31, 38, 46], and improving the health and happiness of nursing employees [36, 37, 43, 50]. Therefore, we should start from the level of nursing leaders and nurse employees, take health values, health awareness, and health behaviors as dimensions, integrate these characteristics and attributes of health-related leadership, and build a specific concept of health leadership in the field of nursing.

### 5.2. Assessment Tools

Many leadership measurement tools are mentioned in the literature reviewed in this study, but their attributes or assessment content are different (see Table 3). Therefore, researchers should fully clarify the measured leadership style or content and attributes when selecting leadership measurement tools [39]. Some measurement tools are specifically used to measure specific leadership styles, such as Health-Promoting Leadership [50], ALQ [29–31, 33, 45, 49], Ethical Leadership Scale (ELS) [43], Survey of Transformational Leadership [39], and they have certain specificity. Some measurement tools do not clearly define what leadership style is to be measured, such as the MLQ [24, 25, 35, 39] and the iLead Scale [25, 28, 35, 39, 51], so they apply to a wide range of leadership. In addition, researchers should also pay attention to the existence of various forms of Authentic Leadership Questionnaire (such as Authentic Leadership Inventory (ALI) [31], Authentic Nurse Leadership Questionnaire (ANLQ) [38, 39, 46], ALQ [29–31, 33, 45, 49]) and MLQ (such as MLQ [24, 25, 35, 39], Multifactor Leadership Questionnaire 5X Short Form [28, 37], Multifactor Leadership Questionnaire Form—6S [51]), but the measurement content, questionnaire details, or psychological characteristics of different forms are different. Therefore, researchers should also consider various aspects when choosing the tool with the best fit for their research. Some measurement tools do not have specific leadership style requirements but mainly measure a single attribute or specific content of leadership, such as measuring leadership behavior or leadership quality, for example, Copenhagen Psychosocial Questionnaire (COPSOQ) [47], Supportive Leadership Behavior Scale [39], and Leader Behavior Description Questionnaire (LBDQ) [23, 28], and their psychological characteristics are good. A measurement tool for health leadership, though, Franke et al. developed and tested a new tool to measure health-promoting leadership but lacked evidence of its widespread use and measurement in nursing [10, 54]. Therefore, assessment tools that promote leadership or do not specify leadership style are a good choice [50]. To sum up, although some of these leadership measurement tools are specific to leadership style, their application fields are wide. Thus, there is a lack of specific tools to measure health leadership style in the field of nursing [39, 54]. In the future, it will be necessary to develop an assessment tool based on the theoretical framework of health leadership in nursing, focusing on measuring health leadership in nursing.

### 5.3. Factors and Well-Being

**Figure 4:**
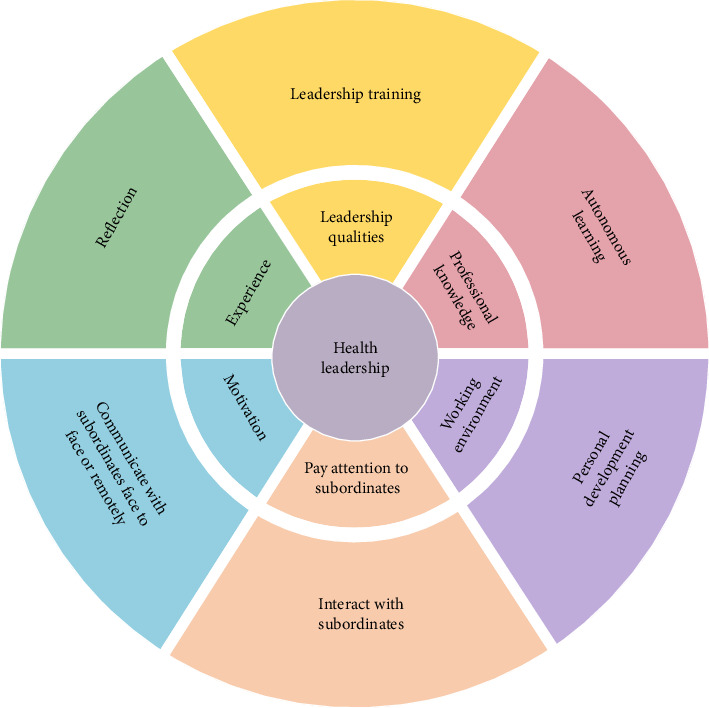
Influencing factors for nursing health leadership and an intervention model (the inner segments are influences, and the outer segments are interventions).

In this study, we identified factors influencing health leadership in the nursing field. These factors exist at the personal and organizational levels. Studies have shown that among the personal factors, the quality of leaders, experience, professional knowledge, leadership style, patience with employees and work, motivation, individualized consideration of employees, and a high tolerance for uncertain behavior (the ability to tolerate nurse uncertainty and delay without anxiety or upset) can influence health leadership through leaders' healthy behaviors [18, 23, 25, 32, 36, 37, 42, 44, 47–51]. If leaders care for and respect nurses, nurses have higher work attendance, better health status, less irritability, health complaints, and work conflicts [18], indicating that leaders' health awareness has a positive impact on leaders' implementation of health leadership to promote nurses' health [18, 44, 45]. Many studies support this view and believe that good personal qualities such as initiative, courage, and personal professional knowledge and experience of leaders are also important factors in promoting the implementation of health leadership, to further improve nurses' job satisfaction [30, 32, 36, 44, 47, 51]. At the same time, healthy leadership behaviors such as individualized consideration for nurses and occasional rewards at work can promote the personal accomplishment of nurses and effectively reduce emotional exhaustion [23, 25]. From the perspective of nurses, they also prefer leaders with consideration, persuasion skills, and tolerance for uncertain behavior [23]. It can also be said that when leaders have leadership skills and exhibit a positive and supportive leadership style, their ability to promote health increases, such as increasing nurses' job satisfaction and willingness to stay in their posts [37, 42, 49]. However, when leaders face high-performance pressure, they will also increase the workload of subordinates to meet high-level job requirements, which weakens the negative impact of health-promoting leadership on subordinates' workload [50]. In addition, taking leaders' self-awareness and psychological capital as mediating variables, they play a mediating role between leadership and job engagement [22]. For example, leaders pay less attention to health, and a lack of health promotion commitment may hinder leaders from implementing health leadership [32]. Among organizational factors, a healthy working environment is considered to be a promoter of healthy behaviors [32], which not only helps leaders to develop health leadership but also promotes a healthy way for nursing employees to behave [18, 32, 48]. In a positive organizational culture, nurse managers express optimism and personal satisfaction with their position, helping them to create their environment, which has a positive impact on staff, patients, and the organization [26]. According to the research, maintaining the transparency of organizational relationships [42, 45] and providing nurses with support and rewards [25, 30] can help to recognize and understand the pressure and needs of nurses, promote the positive psychological state of nurses in the organization, and improve the healthy leadership behavior of leaders [42]. If the organization pays attention to the employees [13, 34, 44], for example, the organization manager is aware of the stress level and poor working conditions of the employees and takes measures to solve them, the significance of the nurses' work will be satisfied and the happiness of the employees will be further improved [28, 31, 37]. As the three dimensions of health leadership [10, 54], health values, health awareness, and health behaviors are positively related to physical and mental health, the satisfaction of leaders and employees, and organizational performance, whereas employee turnover, burnout, and depersonalization are negatively correlated [22, 36, 43, 45], but within the team does not have this role [55]. The results of this study are supported by international literature, which holds that social environment is a determinant of nurses' well-being and that healthy and effective leadership can directly affect nurses' well-being and improve nursing performance and nursing outcomes by regulating the quality of the work environment to improve nurses' perception of the organizational environment [53, 56]. At the same time, the leadership of nurse leaders indirectly affects patients' cognition of nursing work and improves patients' health outcomes through nurses' organizational environment, leadership ability, and behavioral traits [6]. When leaders have health leadership, they can identify and solve employees' health problems and effectively implement work processes [13, 30, 35, 38, 46, 57]. However, many factors influence health leadership, these findings provide evidence for the necessity of improving the health leadership of nursing leaders in the future and the formulation of intervention strategies. We summarized the influencing factors and intervention models of health leadership in the nursing field as shown in Figure 4.

### 5.4. Interventions

The literature on health leadership interventions reviewed in this study is limited. There are some similar measures for improving health leadership and other styles of leadership; for example, they all include self-directed learning and reflection, and online or face-to-face communication with nurses [35, 40, 44]. Improving the psychological level of nursing leaders, participating in nursing practice, and creating a learning space are unique measures of health leadership [40, 44], while personal development plans and leadership training apply to improving leadership in different leadership styles [30, 35]. Our study findings revealed that a series of leadership interventions have a beneficial effect on leadership behavior among nursing leaders [35]. Personal development, self-directed, and reflective intervention programs can promote more frequent leader–employee interaction, more effective implementation processes, and improvements in collaborative communication, problem-solving, and nursing leadership [35, 40]. Beyond this, behavioral interventions by leaders, self-learning, and communication with nurses appear to be some of the most promising measures for promoting a healthy work environment and addressing mental health issues among caregivers [30, 35, 40]. However, research on leadership interventions to maintain or promote mental health remains inadequate and needs to be supplemented by high-quality research designs [40]. Communication with nurses is often cited as an effective intervention and can be used in a variety of ways to communicate with nurses face-to-face and remotely, such as group-based seminars, lectures, coaching, 360° feedback, group discussions, team activities, plenary sessions, role-playing, demonstrations, and simulation exercises, while improving leadership, which effectively improve leaders' and employees' mental health, professional self-efficacy, and employee engagement [35, 44] (Figure 4). In addition, leaders can also ensure that they are prepared for leadership roles by training nurses and creating learning spaces for nurses, helping to promote a supportive work environment while having access to the practical support they need [44]. Whether leaders can adopt these strategies is related to their identity, their sense of self, and their concern for their well-being [44]. Despite this, there is a lack of high-quality research exploring the training effects of health leadership interventions [40], and we urgently need complete and effective health leadership interventions to improve employee engagement and satisfaction, reduce attrition, and achieve organizational vision [40]. In the future, leadership action plans should be incorporated into regular education, and leadership implementation frameworks should be constructed to realize feasible healthy leadership strategies.

## 6. Conclusions

In this scoping review, the concept of health leadership in nursing is clarified. Health leadership is divided into leader-centered and staff-centered leadership, which is based on health values, health awareness, and health behavior. Leaders focus on engagement, positive communication, creating a healthy work environment, and maintaining the physical and mental health of themselves and their employees through leadership attributes such as caring, support, ethics, compassion, courage, and responsibility. Twenty-four evaluation tools for health leadership have been found, among which the most relevant one is Health-Promoting Leadership Conditions, which can be used to evaluate the health promotion leadership of nursing managers. However, tools to assess health leadership in the nursing field need to be further developed. Health leadership is also influenced by personal and organizational factors, where leadership qualities, specific knowledge, self-awareness, psychology, leadership style, motivation, consideration, and a commitment to health issues are common personal factors. Organizational factors include the work environment, attention to subordinates, job expectations, and relationship transparency. Identifying these factors can provide a reliable basis for developing tools to assess and improve health leadership in the nursing field. The findings of this study also suggest that personal development planning, leadership training, face-to-face communication, self-directed learning, and reflection are effective interventions for promoting health leadership and improving the well-being of nursing leaders and staff members. In the future, a variety of interventions can be implemented to improve the health leadership of care managers and bring health and well-being to caregivers.

## 7. Limitations of the Study

This study has certain limitations. Only electronic databases and journals with high scientific value were considered in this study, but this may also cause some relevant articles to go undiscovered. In some of the included studies, the sample population included people working in the medical field (and nursing field). Because these studies were not conducted solely for individuals employed in the nursing field, the specificity of the results was limited. We only considered studies published in English and could have excluded relevant studies in other languages and countries. In addition, the definition of the role of nurses may differ in different countries. The study aim was the field of nursing, which includes nurses in a broad sense. Due to the limited definition of health leadership articles, the concept of health leadership was broadened and not limited to a single conceptual theory during the literature review process, and it includes leadership behaviors with health as a long-term goal; however, the consensus reached among researchers was satisfactory. In the future, we should explore and construct a more specific and optimized intervention framework, develop training strategies and assessment tools for nursing leaders, and conduct well-designed randomized controlled trials to provide high-quality research evidence while continuously improving health leadership in the field of nursing.

## 8. Implications for Nursing Management

This study provides useful knowledge and insights that can motivate nursing managers to consciously incorporate health leadership styles into nursing practice to continuously focus on and improve their own and their subordinates' health. Health leadership is considered a leadership style that contributes to the health and well-being of nursing managers, nursing staffs, organizations, and patients, creates a positive and healthy work environment, maintains the physical and mental health of managers, promotes the well-being and satisfaction of nursing staffs, and improves patient health outcomes. Therefore, nursing managers should clarify the importance of health leadership, constantly strengthen the training of health leadership, and promote the establishment of sustainable healthcare teams.

## Figures and Tables

**Table 1:** Mapping of study characteristics of all studies included in this review.

Study characteristics (*N* = 32)	*N* (%)
Publication years	
2008–2013	4 (12.50)
2014–2018	9 (28.12)
2019–2023	19 (59.38)
Country of origin (top 5)	
USA	5 (15.63)
Germany	5 (15.63)
Canada	4 (12.50)
Australian	7 (21.88)
Finland	2 (6.25)
Study types	
Review	3 (9.37)
Cohort study	1 (3.13)
Systematic review	5 (15.62)
Cross-sectional study	16 (50.00)
Qualitative study	4 (12.50)
Scoping review	2 (6.25)
Quasi-experimental and mixed methods	1 (3.13)

**Table 2:** Basic information on health leadership.

Author	Country	Type of study	Sample size	Research topic (conceptual characteristics)	Instrumentation	Correlation factors	Interventions	Health effects
Shirey [24]	USA	Review		Authentic leadership (confident, optimistic, resilient, transparent, ethical, future-oriented.)	Servant Leadership Behavior Scale Multifactor Leadership Questionnaire (MLQ)			Healthy working environment (+)

Kanste [25]	Finland	A cross-sectional study	*N* = 627	Leadership behavior	Multifactor Leadership Questionnaire (MLQ)	Personal factors: Inspirational motivation (+) Intellectual motivation (+) Consideration (+) Organizational factors: Contingent reward (+)		Personal accomplishment (+) Mental health (emotional exhaustion) (−) Depersonalization (−)

Shirey [26]	USA	Qualitative descriptive study	*N* = 21	Authentic leadership (confident, optimistic, resilient, transparent, ethical, future-oriented.)		Organizational factors: Organizational culture (+)		Healthy working environment (+)

Lee et al. [27]	Canada	Quasi-experimental and mixed methods	*N* = 86	Leadership (mutual respect, motivation, inspiring others, dynamic processes.)	Leadership Practices Inventory (LPI)			

Gregersen, Vincent-Hoper, and Nienhaus [28]	Germany	A cross-sectional study	*N* = 1045	Transformational leadership (high credibility, exemplary character, attractive vision, the ability to motivate employees, provision of support and development.)	Leader Behavior Description Questionnaire (LBDQ) Multifactor Leadership Questionnaire 5X Short Form (MLQ 5X Short Form) LMX 7-scale			Well-being (+)

Read and Laschinger [29]	Canada	A cross-sectional study	*N* = 191	Authentic leaders (confident, optimistic, resilient, transparent, ethical, future-oriented.)	Authentic Leadership Questionnaire (ALQ)			A healthy work environment (+) Job satisfaction (+) Mental health (+)

Laschinger et al. [30]	Canada	A cross-sectional study	*N* = 1009	Authentic leadership (confident, optimistic, resilient, transparent, ethical, future-oriented.)	Authentic Leadership Questionnaire (ALQ)	Organizational factors: Job expectations (+) Workload (−) Control (+) Rewards (+)	Leadership skills training	Burnout (−) Mental health (+) Healthy working environment (+) Professional self-efficacy (+)

Furunes, Kaltveit, and Akerjordet [13]	Australia	Qualitative study	*N* = 12	Health‐promoting leadership (focusing on participation, meaningful work, skills development, being caring, supportive, courageous, responsible, attentive, and ethical.)		Organizational factors: Attention to subordinates (+)		Health‐promoting work environment (+)

Akerjordet, Furunes, and Haver [18]	Australia	An integrative review		Health‐promoting leadership (focusing on participation, meaningful work, skills development, being caring, supportive, courageous, responsible, attentive, and ethical.)		Personal factors: Leadership qualities (+) Consideration (+) Organizational factors: Work environment (+) Focus on participation (+)		

Bansal and Malhotra [23]	India	A cross-sectional study	*N* = 621	Leadership behavior	Leader Behavior Description Questionnaire (LBDQ)	Personal factors: Consideration (+) Persuasion skills (+) Tolerance of uncertain behavior (+)		Job satisfaction (+)

Malila, Lunkka, and Suhonen [31]	Finland	A scoping review		Authentic leadership (confident, optimistic, resilient, transparent, ethical, future-oriented.)	Authentic Leadership Questionnaire (ALQ) Authentic Leadership Inventory (ALI) Leadership Practices Inventory (LPI)			Well-being (+) Healthy working environment (+)

Alilyyani, Wong, and Cummings [22]	Canada	A systematic review		Authentic leadership (confident, optimistic, resilient, transparent, ethical, future-oriented.)		Personal factors: Psychological capital (+)		Mental health (+) Satisfaction (+) Burnout (−)

Horstmann and Remdisch [32]	Germany	Qualitative study	*N* = 51	Health-specific leadership (leader-centered and staff-centered, health values, health awareness, health behaviors, creating working conditions, promoting mental health, communication, and interaction.)		Personal factors: Leadership qualities (+) Specific knowledge (+) Commitment to health issues (+) Organizational factors: Organizational vision (+)		

Anwar, Abid, and Waqas [33]	Pakistan	A cross-sectional studies	*N* = 172	Authentic leadership (confident, optimistic, resilient, transparent, ethical, future-oriented.)	Authentic Leadership Questionnaire (ALQ)			Nurses' desire for creativity (+)

Worringer et al. [34]	Germany	Qualitative study	*N* = 37	Health-oriented supportive (leader-centered and staff-centered, health values, health awareness, health behaviors, creating working conditions, promoting mental health, communication, and interaction.)		Organizational factors: Pay attention to subordinates (+)		Mental health (work pressure) (−) Physical and mental health (+)

Cleary et al. [35]	Australia	A systematic review		Leadership (mutual respect, motivation, inspiring others, dynamic processes.)	Multifactor Leadership Questionnaire (MLQ) ICU Nurse–Physician Questionnaire Modified Nurse Manager Inventory Tool Leadership Practices Inventory (LPI)		Develop a personal development plan; Communicate with nurses online/face to face; self-directed learning and reflection; respond to the needs of individuals and organizations.	Implementation of efficient processes (+) Staff engagement (+) Satisfaction (+)

Dyrbye et al. [36]	USA	A cross-sectional study	*N* = 3989 6	Leadership behavior		Personal factors: Leadership qualities (+)		Burnout (−) Satisfaction (+)

Sabbah et al. [37]	Lebanese	A cross-sectional study	*N* = 250	Leadership (mutual respect, motivation, inspiring others, dynamic processes.)	Multifactor Leadership Questionnaire 5X Short Form (MLQ 5X Short Form) SF-12v2 Health Survey (SF-12v2)	Personal factors: Leadership styles (+)		Well-being (+)

Raso, Fitzpatrick, and Masick [38]	USA	A cross-sectional study	*N* = 254	Authentic leadership (confident, optimistic, resilient, transparent, ethical, future-oriented.)	Authentic Nurse Leadership Questionnaire (ANLQ) Critical Elements of a Healthy Work Environment Survey (CE-HWES)			Healthy working environment (+)

Carlson et al. [39]	Australia	A systematic review		Leadership (mutual respect, motivation, inspiring others, dynamic processes.)	Authentic Leadership Self-Assessment Questionnaire iLead scale Spiritual Leadership Questionnaire Supportive Leadership Behaviors Scale Survey of Transformational Leadership Evidence-Based Nursing Leadership Scale Implementation Leadership Scale Authentic Nurse Leadership Questionnaire (ANLQ) Multifactor Leadership Questionnaire (MLQ)			

Stuber et al. [40]	Germany	A systematic review		Health-oriented leadership (leader-centered and staff-centered, health values, health awareness, health behaviors, creating working conditions, promoting mental health, communication, and interaction.)			Mental health Education; Set up education, reflection, and practice groups.	Mental health (+)

Fennell [41]	Australia	A scoping review		Leadership (mutual respect, motivation, inspiring others, dynamic processes.)				

Specchia et al. [42]	Italy	A systematic review		Leadership (mutual respect, motivation, inspiring others, dynamic processes.)		Personal factors: Motivation (+) Self-awareness (+) Work ethic (+) leadership style (+) Organizational factors: Transparency (+)		Job satisfaction (+)

Franczukowska et al. [43]	Australia	A cross-sectional study	*N* = 458	Ethical leadership (personal qualities, interpersonal relationship, selflessness, integrity, fairness, care, and support for subordinates.)	Ethical Leadership Scale (ELS)			Job satisfaction (+) Emotional commitment (+) Burnout (−)

Dickson et al. [44]	UK	Review		Healthful leadership (being visible and present, being open and engaging, caring for self and others, embodying values, being prepared and preparing others, using available information and support, compassion, empathy the values of heart, courage, and authenticity.)		Personal factors: Support (+) Care (+)	Face-to-face/remote communication with nurses; Create a learning space.	

Almeida and Miclos [45]	Brazil	A cross-sectional study	Leadership (*N* = 12) Subordinates (*N* = 180)	Authentic leadership (confident, optimistic, resilient, transparent, ethical, future-oriented.)	Authentic Leadership Questionnaire (ALQ)	Personal factors: Self-awareness (+) Organizational factors: Relationship transparency (+)		Leader: There is no evidence of a relationship with burnout. Subordinate: Negatively related to job burnout.

Raso, Fitzpatrick, and Masick [46]	USA	A cross-sectional study	*N* = 1795	Authentic leadership (confident, optimistic, resilient, transparent, ethical, future-oriented)	Authentic Nurse Leadership Questionnaire (ANLQ)			Healthy work environment (+)

Kuchenbaur and Peter [47]	Germany	Cohort study	*N* = 231	Leadership behavior	Copenhagen Psychosocial Questionnaire (COPSOQ)	Personal factors: Leadership qualities (+) Effort–reward imbalance (+) Support (+)		Physical and mental health (+)

Giallouros et al. [48]	Cyprus	A cross-sectional study	*N* = 605	Leadership (mutual respect, motivation, inspiring others, dynamic processes.)		Personal factors: Leadership role encouragement (+) Organizational factors: Shared organizational vision (+)		

Al-Hassan et al. [49]	Jordan	A cross-sectional study	*N* = 170	Authentic leadership (confident, optimistic, resilient, transparent, ethical, future-oriented.)	Authentic Leadership Scale (ALQ)	Personal factors: Leadership style (+)		Bullying and burnout (−)

Shan et al. [50]	China	A cross-sectional study	*N* = 98	Health-promoting leadership (focuing on participation, meaningful work, skills development, being caring, supportive, courageous, responsible, attentive, and ethical.)	Health-Promoting Leadership Conditions	Personal factors: Pressure of nursing leaders (−)		Burnout (−)

Middleton et al. [51]	Australia	A cross-sectional study	*N* = 80	Leadership (mutual respect, motivation, inspiring others, dynamic processes.)	Multifactor leadership questionnaire Form—6S	Personal factors: Experience (+)		

*Note:* “+” positive correlation, “−” negative correlation.

**Table 3:** Basic information on the nursing field leadership scale.

Scale name	Measures	Scale details	Reliability
Health-Promoting Leadership [50]	The health-promoting leadership of chief nurses.	• 21 items. • Likert (7-point). • Seven dimensions: health awareness, workload, control, reward, community, fairness, and value-fit.	Cronbach's alpha = 0.98. Cronbach's alpha range for the seven dimensions of the scale was 0.91–0.98.

Copenhagen Psychosocial Questionnaire (COPSOQ) [47]	Quality of leadership, work–privacy conflict.	• Likert (5-point).	Quality of leadership (Cronbach's alpha = 0.92, original: Cronbach's alpha = 0.89). Work–privacy conflict (Cronbach's alpha = 0.83, original: Cronbach's alpha = 0.90).

Leader Behavior Description Questionnaire (LBDQ) [23, 28]	Leadership behavior.	• 16 items for consideration and 12 items for initiating. • Likert (5-point).	Cronbach's alpha = 0.948.

Multifactor Leadership Questionnaire 5X Short Form [28, 37]	Staff nurses' opinions of their nurse managers.	• 45 items. • Likert (5-point). • Nine characteristics of transformational, transactional, or passive/avoidant leadership styles and three outcomes of leadership behaviors, which are extra effort (EF), effectiveness (EFF), and satisfaction (SAT).	Cronbach's alpha = 0.73∼0.94.

LMX 7 scale [28]	Assess leader–Member exchange.	• 7 items. • Likert (5-point).	Not mentioned.

Multifactor Leadership Questionnaire (MLQ) [24, 25, 35, 39]	Both leader self-evaluation and subordinate evaluation of their supervisor's transformational, active, and passive leadership attributes.	• 78 items. • Likert (5-point). • Nine domains: idealized influence (attributed); idealized influence (behavior); inspiration motivation; intellectual stimulation; individualized consideration; contingent reward; active management-by-exception; passive management-by-exception; and laissez-faire leadership.	Face validity (Y) Content validity (Y) Construct validity (Y) Internal consistency (Y only subscales reported)

Multifactor Leadership Questionnaire Form—6S [51]	Leadership style.	• 21 items. • Likert (5-point). • The seven factors within the scale are then grouped into three leadership styles (transformational, transactional, and passive avoidant).	Cronbach's alpha = 0.78.

Authentic Leadership Questionnaire (ALQ) [29–31, 33, 45, 49]	Authentic leadership behavior.	• 16 items. • Likert (5-point). • Four dimensions of authentic leadership behavior: self-awareness (4 items), moral-ethical perspective (4 items), balanced processing (3 items), and transparency (5 items).	Cronbach's alpha = 0.96.

Authentic Leadership Self-Assessment Questionnaire [39]	Self-evaluated authentic leadership.	• 16 items. • Likert (5-point). • Four domains: self-awareness; internalized moral perspective; balanced processing; relational transparency.	Content validity (Y) Construct validity (Y) Internal consistency (Y)

Authentic Nurse Leadership Questionnaire (ANLQ) [38, 39, 46]	Self-evaluated authentic leadership in nurse leaders.	• 29 items. • Five domains: self-awareness; moral ethical; relational integrity; shared decision-making; caring.	Cronbach's alpha = 0.97.

The iLead Scale [39]	Employees' perspectives on their managers' active and passive implementation-specific leadership attributes.	• 20 items. • Likert (5-point). • Two domains: active leadership and passive leadership. • Active leadership subdomains: exemplary behavior, individualized consideration, intellectual stimulation, and contingent reward. • Passive leadership subdomains: passive management-by-exception and laissez-faire.	Face validity (Y) Content validity (Y) Criterion validity (Y) Internal consistency (Y)

Spiritual leadership questionnaire [39]	Nurses' spiritual leadership attributes.	• 35 items. • Likert (5-point). • Eight domains: vision: faith and hope; altruism; inner life; calling; membership; organizational commitment; and productivity.	Face validity (Y) Content validity (Y) Internal consistency (Y) Test-retest reliability (Y)

Supportive Leadership Behavior Scale [39]	Supportive leadership behavior.	• 20 items. • Likert (5-point). • Four domains: support for development; integrity; sincerity; and recognition	Face validity (Y) Content validity (Y) Construct validity (Y) Internal consistency (Y) Test–retest reliability (Y)

Survey of Transformational Leadership [39]	Approaches to the conceptualization and measurement of transformational practices.	• 84 items. • Likert (5-point). • Five domains: idealized influence; intellectual stimulation; inspirational motivation; individualized consideration; and empowerment.	Face validity (Y) Content validity (Y) Construct validity (Y) Criterion validity (Y) Internal consistency (Y)

Evidence-Based Nursing Leadership Scale [39]	Staff nurse perceptions of support provided by unit-level nurse managers to engage in EBP.	• 10 items. • Response scale not specified. • Item examples: “My manager provides time for me to engage in EBP” and “My manager makes sure that I have access to relevant research on my unit.”	Face validity (Y) Content validity (Y) Construct validity (Y) Internal consistency (Y)

Implementation Leadership Scale [39]	Clinician reports of strategic leadership attributes in supervisors, specific to EBP implementation.	• 12 items. • Likert (5-point). • Domains: proactive leadership; knowledgeable leadership; supportive leadership; and perseverant leadership.	Face validity (Y) Content validity (Y) Construct validity (Y) Internal consistency (Y)

ICU Nurse–Physician Questionnaire [35]	Significant improvement at follow-up in self-perceived leadership as well as leadership and communication skills satisfaction.	Not mentioned	Not mentioned

Modified Nurse Manager Inventory Tool [35]	Leadership intervention behavior.	Not mentioned	Not mentioned

Critical Elements of a Healthy Work Environment Survey (CE-HWES) [38]	The health of the work environment in the participants' work units and their organizations.	• 32 items. • Likert (4-point). • For this study, only the 16-item work unit scale was used.	Cronbach's alpha = 0.93.

Servant Leadership Behavior Scale [24]	Measure the authenticity factor of authentic leadership.	Not mentioned	Not mentioned

Leadership Practices Inventory (LPI) [27, 31, 35]	Original LPI is recommended when used as an educational tool.	Original LPI: • 30 items. • Likert (5-point). • Five domains: challenging the process; inspiring a shared vision; enabling others to act; modeling the way; and encouraging the heart.	Content validity (Y) Construct validity (Y) Internal consistency (Y)

SF-12v2 Health Survey (SF-12v2) [37]	Measure the overall state of life.	• 12 items. • Eight domains: physical functioning (PF); physical role (RP); bodily pain (BP); general health (GH); vitality (VT); social functioning (SF); emotional role (RE); and mental health (MH).	Not mentioned

Ethical Leadership Scale (ELS) [43]	Assessing ethical leadership.	• 10 items.	Cronbach's alpha > 0.779, detailed figures were not mentioned.

Authentic Leadership Inventory (ALI) [31]	Employee's perspectives on their managers' authentic leadership characteristics.	14 items. • Likert (5-point). • Five domains: self-awareness; balanced processing; moral/ethical behavior; and relational transparency.	Content validity (Y) Construct validity (Y) Internal consistency (Y)

*Note:* Reliability refers to surface validity/content validity/construct validity/internal consistency/retest reliability mentioned in the article. “Y” means that the reliability of the scale mentioned in the article meets the requirements, such as Cronbach's alpha > 0.7, but the specific data are not elaborated in the article.

## Data Availability

The data used to support the findings of this study are included within the article.

## References

[1] Liu J. X., Goryakin Y., Maeda A., Bruckner T., Scheffler R. (2017). Global Health Workforce Labor Market Projections for 2030. *Human Resources for Health*.

[2] Zhang X., Tai D., Pforsich H., Lin V. W. (2018). United States Registered Nurse Workforce Report Card and Shortage Forecast: A Revisit. *American Journal of Medical Quality*.

[3] Burmeister E. A., Kalisch B. J., Xie B. (2019). Determinants of Nurse Absenteeism and Intent to Leave: An International Study. *Journal of Nursing Management*.

[4] Pishgooie A. H., Atashzadeh-Shoorideh F., Falcó-Pegueroles A., Lotfi Z. (2019). Correlation Between Nursing Managers’ Leadership Styles and Nurses’ Job Stress and Anticipated Turnover. *Journal of Nursing Management*.

[5] Guo X., Xiong L., Wang Y. (2022). Chinese Nurses’ Perceptions on Toxic Leadership Behaviours of Nurse Managers: A Qualitative Study. *Journal of Nursing Management*.

[6] Zaghini F., Fiorini J., Piredda M., Fida R., Sili A. (2020). The Relationship between Nurse Managers’ Leadership Style and Patients’ Perception of the Quality of the Care provided by Nurses: Cross Sectional Survey. *International Journal of Nursing Studies*.

[7] Efimov I., Harth V., Mache S. (2021). Health Promotion in Virtual Teamwork Through Health-Oriented Leadership. *Prävention und Gesundheitsförderung*.

[8] Mazzetti G., Vignoli M., Petruzziello G., Palareti L. (2018). The Hardier You Are, the Healthier You Become. May Hardiness and Engagement Explain the Relationship Between Leadership and Employees’ Health?. *Frontiers in Psychology*.

[9] Yao L., Li P., Wildy H. (2021). Health-Promoting Leadership: Concept, Measurement, and Research Framework. *Frontiers in Psychology*.

[10] Horstmann D., Eckerth H. L., Wiencke M., Cacace M., Fischer S. (2016). The Need for Healthy Leadership in the Health Care Sector: Consideration of Specific Conditions for Implementation. *Healthy at Work: Interdisciplinary Perspectives*.

[11] Cummings G. G., Tate K., Lee S. (2018). Leadership Styles and Outcome Patterns for the Nursing Workforce and Work Environment: A Systematic Review. *International Journal of Nursing Studies*.

[12] Niinihuhta M., Häggman-Laitila A. (2022). A Systematic Review of the Relationships Between Nurse Leaders’ Leadership Styles and Nurses’ Work-Related Well-Being. *International Journal of Nursing Practice*.

[13] Furunes T., Kaltveit A., Akerjordet K. (2018). Health-Promoting Leadership: A Qualitative Study From Experienced Nurses’ Perspective. *Journal of Clinical Nursing*.

[14] Jiménez P., Bregenzer A., Kallus K. W., Fruhwirth B., Wagner-Hartl V. (2017). Enhancing Resources at the Workplace With Health-Promoting Leadership. *International Journal of Environmental Research and Public Health*.

[15] Skarholt K., Blix E. H., Sandsund M., Andersen T. K. (2016). Health Promoting Leadership Practices in Four Norwegian Industries. *Health Promotion International*.

[16] Frangieh J., Jones T. (2022). Factors Facilitating or Inhibiting the Capacity for Effective Leadership Among Front-Line Nurse Managers: A Scoping Review. *Journal of Nursing Management*.

[17] Efimov I., Harth V., Mache S. (2020). Health-Oriented Self- and Employee Leadership in Virtual Teams: A Qualitative Study With Virtual Leaders. *International Journal of Environmental Research and Public Health*.

[18] Akerjordet K., Furunes T., Haver A. (2018). Health-Promoting Leadership: An Integrative Review and Future Research Agenda. *Journal of Advanced Nursing*.

[19] Colquhoun H. L., Levac D., O’Brien K. K. (2014). Scoping Reviews: Time for Clarity in Definition, Methods, and Reporting. *Journal of Clinical Epidemiology*.

[20] Tricco A. C., Lillie E., Zarin W. (2018). PRISMA Extension for Scoping Reviews (PRISMA-ScR): Checklist and Explanation. *Annals of Internal Medicine*.

[21] Peters M. D. J., Marnie C., Tricco A. C. (2020). Updated Methodological Guidance for the Conduct of Scoping Reviews. *JBI Evidence Synthesis*.

[22] Alilyyani B., Wong C. A., Cummings G. (2018). Antecedents, Mediators, and Outcomes of Authentic Leadership in Healthcare: A Systematic Review. *International Journal of Nursing Studies*.

[23] Bansal R. N., Malhotra M. (2018). Catalyzing Public Healthcare Efficacy: Striking the Right Cords of Leadership. *Journal of Clinical and Diagnostic Research*.

[24] Shirey M. R. (2006). Authentic Leaders Creating Healthy Work Environments for Nursing Practice. *American Journal of Critical Care*.

[25] Kanste O. (2008). The Association Between Leadership Behaviour and Burnout Among Nursing Personnel in Health Care. *Nordic Journal of Nursing Research*.

[26] Shirey M. R. (2009). Authentic Leadership, Organizational Culture, and Healthy Work Environments. *Critical Care Nursing Quarterly*.

[27] Lee H., Spiers J. A., Yurtseven O. (2010). Impact of Leadership Development on Emotional Health in Healthcare Managers. *Journal of Nursing Management*.

[28] Gregersen S., Vincent-Hoper S., Nienhaus A. (2014). Health-Relevant Leadership Behaviour: A Comparison of Leadership Constructs. *German Journal of Human Resource Management: Zeitschrift für Personalforschung*.

[29] Read E. A., Laschinger H. K. S. (2015). The Influence of Authentic Leadership and Empowerment on Nurses’ Relational Social Capital, Mental Health and Job Satisfaction Over the First Year of Practice. *Journal of Advanced Nursing*.

[30] Laschinger H. K., Borgogni L., Consiglio C., Read E. (2015). The Effects of Authentic Leadership, Six Areas of Worklife, and Occupational Coping Self-Efficacy on New Graduate Nurses’ Burnout and Mental Health: A Cross-Sectional Study. *International Journal of Nursing Studies*.

[31] Malila N., Lunkka N., Suhonen M. (2018). Authentic Leadership in Healthcare: A Scoping Review. *Leadership in Health Services*.

[32] Horstmann D., Remdisch S. (2019). Drivers and Barriers in the Practice of Health-Specific Leadership: A Qualitative Study in Healthcare. *Work*.

[33] Anwar A., Abid G., Waqas A. (2019). Authentic Leadership and Creativity: Moderated Meditation Model of Resilience and Hope in the Health Sector. *European Journal of Investigation in Health, Psychology and Education*.

[34] Worringer B., Genrich M., Müller A., Junne F., Angerer P., Angerer P. (2020). How Do Hospital Medical and Nursing Managers Perceive Work-Related Strain on Their Employees?. *International Journal of Environmental Research and Public Health*.

[35] Cleary M., Kornhaber R., Thapa D. K., West S., Visentin D. (2020). A Systematic Review of Behavioral Outcomes for Leadership Interventions Among Health Professionals. *Journal of Nursing Research*.

[36] Dyrbye L. N., Major-Elechi B., Hays J. T., Fraser C. H., Buskirk S. J., West C. P. (2020). Relationship Between Organizational Leadership and Health Care Employee Burnout and Satisfaction. *Mayo Clinic Proceedings*.

[37] Sabbah I. M., Ibrahim T. T., Khamis R. H. (2020). The Association of Leadership Styles and Nurses Well-Being: A Cross-Sectional Study in Healthcare Settings. *The Pan African medical journal*.

[38] Raso R., Fitzpatrick J. J., Masick K. (2020). Clinical Nurses’ Perceptions of Authentic Nurse Leadership and Healthy Work Environment. *The Journal of Nursing Administration: The Journal of Nursing Administration*.

[39] Carlson M. A., Morris S., Day F. (2021). Psychometric Properties of Leadership Scales for Health Professionals: A Systematic Review. *Implementation Science*.

[40] Stuber F., Seifried-Dubon T., Rieger M. A. (2021). The Effectiveness of Health-Oriented Leadership Interventions for the Improvement of Mental Health of Employees in the Health Care Sector: A Systematic Review. *International Archives of Occupational and Environmental Health*.

[41] Fennell K. (2021). Conceptualisations of Leadership and Relevance to Health and Human Service Workforce Development: A Scoping Review. *Journal of Multidisciplinary Healthcare*.

[42] Specchia M. L., Cozzolino M. R., Carini E. (2021). Leadership Styles and Nurses’ Job Satisfaction. Results of a Systematic Review. *International Journal of Environmental Research and Public Health*.

[43] Franczukowska A. A., Krczal E., Knapp C., Baumgartner M. (2021). Examining Ethical Leadership in Health Care Organizations and Its Impacts on Employee Work Attitudes: An Empirical Analysis From Austria. *Leadership in Health Services*.

[44] Dickson C. A. W., Davies C., McCormack B. (2022). UK Nurses’ and Midwives’ Experiences of Healthful Leadership Practices during the COVID‐19 Pandemic: A Rapid Realist Review. *Journal of Nursing Management*.

[45] Almeida D., Miclos P. V. (2022). Nursing in Primary Health Care: Association between Leadership, Psychological Capital, and Burnout Implications. *Revista Brasileira de Enfermagem*.

[46] Raso R., Fitzpatrick J. J., Masick K. (2022). Perceptions of US Nurses and Nurse Leaders on Authentic Nurse Leadership, Healthy Work Environment, Intent to Leave and Nurse Well-Being during a Second Pandemic Year: A Cross Sectional Study. *Journal of Nursing Management*.

[47] Kuchenbaur M., Peter R. (2023). Quality of Leadership and Self-Rated Health: The Moderating Role of ’Effort-Reward Imbalance’: A Longitudinal Perspective. *International Archives of Occupational and Environmental Health*.

[48] Giallouros G., Nicolaides C., Gabriel E. (2023). Enhancing Employee Engagement Through Integrating Leadership and Employee Job Resources: Evidence From a Public Healthcare Setting. *International Public Management Journal*.

[49] Al-Hassan N. S., Rayan A. H., Baqeas M. H., Hamaideh S. H., Khrais H. (2023). Authentic Leadership and Its Role in Registered Nurses’ Mental Health and Experiences of Workplace Bullying. *SAGE Open Nursing*.

[50] Shan G. Y., Wang W., Wang S. N., Zhang Y. J., Li Y. X. (2023). Cross-level Effects of Health-Promoting Leadership on Nurse Presenteeism: The Mediation and Moderation Effect of Workload and Performance Pressure. *Current Psychology*.

[51] Middleton R., Montgomery A., Murray S., Peters S., Halcomb E. (2023). Exploring Leadership in Health Professionals Following an Industry-Based Leadership Program: A Cross-Sectional Survey. *Journal of Advanced Nursing*.

[52] Eriksson A., Axelsson R., Axelsson S. B. (2011). Health Promoting Leadership-Different Views of the Concept. *Work*.

[53] Fiorini J., Zaghini F., Mannocci A., Sili A. (2022). Nursing Leadership in Clinical Practice, Its Efficacy and Repercussion on Nursing-Sensitive Outcomes: A Cross-Sectional Multicentre Protocol Study. *Journal of Nursing Management*.

[54] Franke F., Felfe J., Pundt A. (2014). The Impact of Health-Oriented Leadership on Follower Health: Development and Test of a New Instrument Measuring Health-Promoting Leadership. *German Journal of Human Resource Management: Zeitschrift für Personalforschung*.

[55] Lee S. E., Hyunjie L., Sang S. (2023). Nurse Managers’ Leadership, Patient Safety, and Quality of Care: A Systematic Review. *Western Journal of Nursing Research*.

[56] Della Bella V., Fiorini J., Gioiello G., Zaghini F., Sili A. (2022). Towards a New Conceptual Model for Nurses’ Organizational Well-Being: An Integrative Review. *Journal of Nursing Management*.

[57] Klug K., Felfe J., Krick A. (2022). Does Self-Care Make You a Better Leader? A Multisource Study Linking Leader Self-Care to Health-Oriented Leadership, Employee Self-Care, and Health. *International Journal of Environmental Research and Public Health*.

